# Pediatric traumatic cataracts: 10-year experience of a tertiary referral center

**DOI:** 10.1186/s12886-022-02427-6

**Published:** 2022-05-02

**Authors:** Nesrin Tutaş Günaydın, Ayşe Yeşim Aydın Oral

**Affiliations:** 1 Department of Ophthalmology, University of Health Sciences, Dr. Lütfi Kırdar Kartal City Hospital, Denizer Cad. No:1, 34865, Cevizli, 34100 İstanbul, Turkey; 2 Department of Ophthalmology, Afyonkarahisar University of Health Sciences, 03200 Afyonkarahisar, Turkey

**Keywords:** Anterior vitrectomy, Pediatric traumatic cataract, Posterior capsulotomy, Visual acuity

## Abstract

**Background:**

This study aimed to evaluate the factors influencing final visual acuity in pediatric traumatic cataracts.

**Methods:**

Data of patients who presented with traumatic cataracts were reviewed retrospectively. We evaluated age at trauma; gender, trauma type, cause, and zone; duration between the time of trauma and cataract surgery; surgical method used; time, location, and type of intraocular lens (IOL) implantation; initial and final best corrected visual acuity (BCVA); amblyopia rate; and complications.

**Results:**

In all, 61 eyes of 59 patients aged < 16 years with cataracts after trauma were included. The mean age of the children was 7.2 ± 3.9 years. Primary IOL implantation was performed in 70.9% of eyes. The BCVA was 0.7 LogMAR or better in 5.9% of the 49 eyes in which the visual acuity could be measured at the time of trauma and in 69.1% of 55 eyes in which it could be measured after treatment. Evaluation of factors potentially influencing the final visual acuity revealed that eyes that had undergone posterior capsulotomy (PC) and anterior vitrectomy (AV) during cataract surgery had significantly better final visual acuity compared with eyes that did not undergo these procedures.

**Conclusions:**

In children with posttraumatic cataracts, final visual acuity was not affected by patient age and gender; trauma type, cause, and zone; duration between the time of trauma and cataract surgery; surgical method used; and time, location, and type of intraocular lens (IOL) implantation. Improvements in the final BCVA could be seen only by PC + AV combined with lens aspiration with or without IOL implantation. However, this approach of amblyopia treatment needs to be confirmed by more comprehensive and prospective studies.

**Supplementary Information:**

The online version contains supplementary material available at 10.1186/s12886-022-02427-6.

## Background

Ocular trauma in childhood is a common cause of preventable vision loss in developing countries, and traumatic cataracts make up 12–46% of all pediatric cataracts [1, 2]. The management of traumatic cataracts in the pediatric population is particularly challenging because of the ongoing growth of the eye, risk for amblyopia, and long life expectancy [3].

Many factors influence the final visual acuity in pediatric cataracts, including the age of the child at the time of trauma, type and cause of the trauma, surgical procedure, and initial visual acuity [4–6]. Recognizing these factors would help develop an appropriate treatment approach for pediatric traumatic cataracts.

In our long-term follow-up study, we investigated the epidemiologic features of pediatric traumatic cataracts and evaluated the factors influencing the final visual acuity in a tertiary clinic. Although there have been conducted long-term follow-up studies on pediatric traumatic cataracts, we believe that our study will provide additional evidence to the existing literature especially on final good visual acuity.

## Methods

In this retrospective study, we analyzed the medical records of children younger than 16 years who had presented at the emergency ward of the Istanbul Dr. Lütfi Kırdar Kartal City Hospital eye clinic with ocular trauma between January 2009 and January 2019 and who subsequently developed a traumatic cataract. All the patient data were obtained from the hospital records, maintained in folders, and cataloged. Hospital record folders were screened by NTG and AYO to collect relevant data and to perform analyses. All patients’ medical history, demographic features, and information regarding surgical procedures, treatment, and follow-up were collected from the hospital records. We included children who developed traumatic cataracts following a penetrating or blunt injury and who had been followed up for at least 1 year. We excluded children with an eye injury caused by an intraocular foreign body and also children whose lens capsules were not intact during primary closure surgery and who had to undergo lens aspiration. All penetrating injury cases underwent primary corneal surgery, followed by secondary cataract surgery. The study was conducted in accordance with the ethical guidelines of the Declaration of Helsinki, and we obtained the necessary permission to conduct this study from the ethics committee of our hospital.

A detailed medical history was obtained from the patients or parents during their first visit and examination for the eye injury. After a suitable wait period for the inflammation to subside, the primary sutures were removed, and cataract surgery was performed. All surgeries were performed by a single experienced surgeon (AYAO). Children younger than 2 years routinely underwent lens aspiration with posterior capsulotomy (PC) and anterior vitrectomy (AV), and a secondary intraocular lens (IOL) implantation was conducted after 2 years of age. PC and AV were always performed with lens aspiration in children aged 8 years or younger [7].

The demographic features of the children, age at the time of trauma, follow-up duration, type and cause of the trauma, surgical interventions, whether PC was performed with AV or not, IOL implantation time (primary or secondary), visual acuity, and complications were recorded. All clinical examinations, including best corrected visual acuity (BCVA) measurement, slit lamp biomicroscopy, intraocular pressure measurement with the Tono-Pen XL (Mentor Ophthalmics, Inc., Norwell, MA, USA), and fundus examination by indirect ophthalmoscopy (or B-scan ultrasonography), were performed at every follow-up examination.

The approach to the cataract surgery, status of IOL implantation, IOL type, and IOL position were noted. The IOL power was determined according to the SRK-T formula before surgery. The intended target refraction was approximately + 3.0 diopters (D) for the 2–4 years age group, + 2.0 D for the 4–6 years age group, + 1.0 D for the 6–8 years age group, and a plano lens for children older than 8 years.

The patients were divided into three groups according to age: Group A, 0–5 years; Group B, 6–10 years; and Group C, 11–15 years. The zone of trauma was defined according to the Ocular Trauma Classification. The patients were accordingly classified as zone 1 (corneal), zone 2 (corneoscleral), or zone 3 (scleral) [8].

The BCVA was evaluated using the Snellen visual acuity chart and Lea symbols as appropriate. All measured visual acuities were converted into logMAR and recorded. The visual acuities were classified into four groups as suggested by Pieramici et al. [8]: Grade 1, 0.3 logMAR or better; Grade 2, 0.7–0.3 logMAR; Grade 3, 1.3–0.7 logMAR; and Grade 4, 1.3 logMAR to light perception. The BCVA at the final follow-up and potential influencing factors were investigated. Our visual outcomes were categorized using the World Health Organization^9^ (WHO) standard, where good vision is 6/6 to 6/18 (0.5 logMAR or better), borderline vision is less than 6/18 to 6/60 (0.5–1.0 logMAR) and poor vision is less than 6/60 (1.0 logMAR or worse) [9].

The relationship between the injury type and the surgical procedure used, in addition to cataract surgery, was also examined in our statistical analyses.

Amblyopia is defined as a decrease in vision of two lines or more in the traumatic eye relative to the non-traumatic eye (e.g. in the Snellen chart). The rates of amblyopia and strabismus and the optic rehabilitation options were also analyzed in the study group. Patients who had amblyopia were regularly followed up with patching. Patching was prescribed starting at the second week after cataract surgery. Caregivers were instructed to have the child wear an adhesive occlusive patch over the fellow eye 1 h daily per month of age until a child was 8 months old. Thereafter, caregivers were told to patch their child 50% of the waking hours, as suggested by the Infant Aphakia Treatment Study Group [10]. Children aged 3 to 7 years with severe amblyopia (from 1.3 logMAR to 0.7 logMAR) were given occlusion therapy for 6 hours a day [11]. In children with moderate amblyopia (from 0.7 logMAR to 0.3 logMAR), occlusion therapy was performed for 2 hours a day [12]. For children aged 7–15 years, occlusion therapy was recommended for 2 hours a day with near visual activities, even if amblyopia had been treated [13].

### Statistical analyses

Statistical analyses were performed with SPSS for Windows (version 22.0; IBM Corp., Armonk, NY, USA). The Mann–Whitney U-test and Kruskal–Wallis test were used for comparison of the independent predictors related to the final BCVA. The chi-square test was used to evaluate the relationship between categorical nominal variables, e.g., posterior capsule opacification (PCO) and PC + AV, injury age range and amblyopia, and injury type and additional surgery. Correlation analyses were made between initial and final BCVA readings using Spearman’s correlation coefficient test and between the time from trauma to cataract surgery and final BCVA using Pearson correlation test. Differences with a *p*-value of less than 0.05 were considered statistically significant.

## Results

Data from hospital records indicated that cataract surgery was performed on 61 eyes from 59 patients for traumatic cataract in the study period. Boys were the more commonly affected of all types of trauma. The age group with the highest rate of traumatic cataracts was Group B (6–10 years old). Demographic characteristics of patients are provided in Table 1. There was no sex- or age-dependent effect on final vision of the patients (Table 2).

**Table 1:** Patient demographics

Variables	TOTAL		Penetrating		Nonpenetrating (Blunt)	
	Mean ± SD	Min–max	Mean ± SD	Min–max	Mean ± SD	Min–max
Injury age (years)	7.2 ± 3.9	0–15	7.3 ± 3.9	0–15	7.5 ± 3.5	2–15
Follow-up duration (years)	3.2 ± 2.1	1–9	3.2 ± 2.2	1–9	3.1 ± 1.7	1–6
	*n*	%	*n*	%	*n*	%
Gender						
Female	17	28.8	11	23.4	6	50
Male	42	71.2	36	76.6	6	50
Total	59	100	47	100	12	100
Age group (years)						
6–10	29	49.2	23	48.9	6	50
0–5	19	32.2	15	31.9	4	33
11–15	11	18.6	9	19.1	2	16
Total	59	100	47	100	12	100
Eye						
Right	30	49.2	23	46.9	7	58,3
Left	31	50.8	26	53.1	5	41.6
Total	61	100	49	100	12	100

**Table 2:** Factors affecting visual acuity after traumatic cataract surgery

	n	Final BCVA (logMAR)		***p***
		Mean ± SD	Median (min–max)	
**Gender (*n*: 59)**				0.258
Female	17	0.84 ± 0.68	0.5 (0.22–2)	
Male	42	0.6 ± 0.5	0.4 (0–2)	
**Age at injury range (*n*: 59)**				0.896
0–5 years	19	0.69 ± 0.53	0.5 (0–2)	
6–10 years	29	0.68 ± 0.61	0.4 (0–2)	
11–15 years	11	0.59 ± 0.48	0.5 (0–1.3)	
**Cause of trauma (*n*: 61)**				0.601
Glass	11	0.57 ± 0.32	0.45 (0.22–1.3)	
Sharp metals	17	0.36 ± 0.13	0.36 (0–0.4)	
Wooden objects	12	0.51 ± 0.34	0.45 (0–1.3)	
Stones	2	0.5 ± 0.42	0.5 (0.2–0.8)	
Firecracker	3	1.03 ± 0.87	0.8 (0.3–2)	
Fingernail	2	0.58 ± 0.29	0.5 (0.4–0.6)	
Blunt trauma	12	0.44 ± 0.41	0.26 (0–13)	
Not known	2	1 ± 0	1 (1–1)	
**Type of injury (*n*: 61)**				0.295
Open globe	49	0.69 ± 0.55	0.45 (0–2)	
Closed globe	12	0.58 ± 0.61	0.3 (0–2)	
**Zone of injury (*n*: 61)**				0.065
Zone 1	31	0.62 ± 0.56	0.4 (0–2)	
Zone 2	13	0.9 ± 0.59	0.7 (0.3–2)	
Zone 3	5	0.49 ± 0.45	0.3 (0–1.3)	
**Type of surgery (*n*: 55)**				0.124
Lens aspiration	13	0.37 ± 0.36	0.31 (0–1)	
Lens aspiration + PCIOL	26	0.66 ± 0.58	0.4 (0–2)	
Lens aspiration + PPV	5	0.86 ± 0.66	0.7 (0.4–2)	
Lens aspiration + membranectomy + pupilloplasty	11	1.03 ± 0.59	1.15 (0.22–2)	
**Lens aspiration + AV + PC (*n*: 55)**				0.048
Performed	42	0.59 ± 0.46	0.4 (0–2)	
Not performed	13	0.77 ± 0.63	0.5 (0.2–2)	
**Time of IOL implantation (*n*: 55)**				0.513
Aphakia	5	0.52 ± 0.59	0.22 (0–1.3)	
Primary	39	0.72 ± 0.57	0.5 (0–2)	
Secondary	11	0.76 ± 0.72	0.4 (0.22–2)	
**IOL implantation location (*n*: 55)**				0.335
Aphakia	5	0.52 ± 0,59	0.22 (0–1.3)	
Bag	19	0.56 ± 0.45	0.4 (0–1.3)	
Sulcus	21	0.83 ± 0.61	0.7 (0.22–2)	
TSF	10	0.9 ± 0.79	0.4 (0.2–2)	
**IOL type (*n*: 50)**				0.467
				0.467
Single-piece foldable IOL	31	0.58 ± 0.41	0.5 (0–1.3)	
Three-piece foldable IOL	19	0.88 ± 0.67	0.8 (0.22–2)	
**Time between injury and cataract surgery (*n*:55)**				0.872
< 3 months	29	0.66 ± 0.53	0.4(0–2)	
> 3 months	26	0.75 ± 0.64	0.5(0–2)	

Mann–Whitney U-test and Kruskal–Wallis tests were used for comparison of the independent predictors related to the final BCVA

*BCVA* Best corrected distance visual acuity, *AV* Anterior vitrectomy, *PC* Posterior capsulotomy, *PPV* Pars plana vitrectomy, *PCIOL* Posterior chamber intraocular lens, *TSF* Trans-scleral fixation

**Fig. 1:**
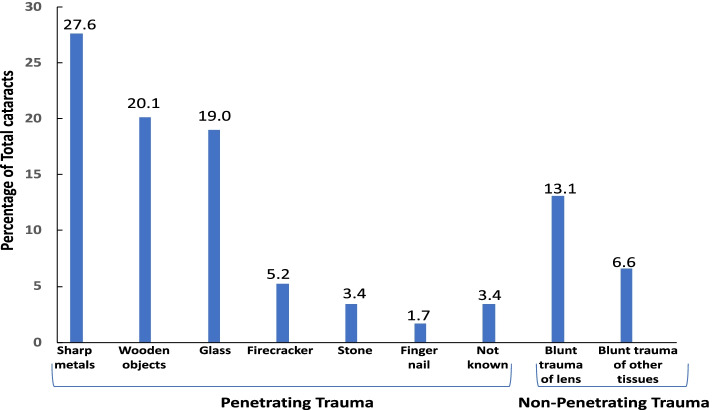
Causes of ocular trauma

The cause of cataract development was penetrating trauma in 49 eyes (80.4%) and blunt trauma in 12 eyes (19.6%). Trauma type had no effect on the final BCVA (*p* = 0.295) (Table 2). The most common cause of trauma was penetrating injury with a sharp object (27.6%) as shown in Fig. 1 and Table 1. Only the lens was affected in 8 of 12 eyes with blunt trauma, whereas 4 eyes had trauma of other tissues besides the cataract. There was no relationship between the cause of trauma (*p* = 0.601) and zone of trauma (*p* = 0.065) on final BCVA (Table 2).

Overall, 6 of the 61 eyes were followed up without cataract surgery, as the initial BCVA was better than 0.3 logMAR, and they were in an age group (10–15 years) with a low risk for amblyopia. Cataract surgery was performed on 55 eyes (90%).

Among the 55 eyes that underwent cataract surgery, 13 (23.6%) were treated with lens aspiration only; 42 (76.4%) eyes underwent PC + AV together with LA. PCO was seen during follow-up in all (100%) of the 13 cases that had undergone lens aspiration without PC + AV, and all of these patients underwent YAG laser capsulotomy. There was a relationship between not performing PC + AV and PCO development (*p* = 0.001). The final BCVA was better in eyes that had undergone PC + AV, compared with those that had not undergone PC + AV (*p* = 0.048) (Table 2).

Among the 55 eyes that underwent cataract surgery, 5 eyes (9.1%) of three patients were followed up as aphakic. A visual acuity of 0.4 logMAR or above (*n* = 3 could be evaluated for BCVA) was achieved, using aphakic glasses in three eyes of three children and aphakic contact lenses in both eyes of a child.

There was no correlation between the time of implantation (whether primary or secondary) (*p* = 0.513), IOL implantation location (*p* = 0.335) and IOL type (*p* = 0.467) had no effect on the final BCVA (Table 2). A significant relationship was found between the type of trauma (blunt or penetrating) and the additional surgical interventions used (*p* = 0.001). Anterior segment interventions such as membranectomy and pupilloplasty were required more often in penetrating cases (*n* = 10, 100%), whereas retinal surgery was required more frequently for blunt injuries (*n* = 3; 25%). Of the 12 patients who had suffered blunt trauma, 11 had cataract surgery, and 3 of them had PPV.

**Fig. 2:**
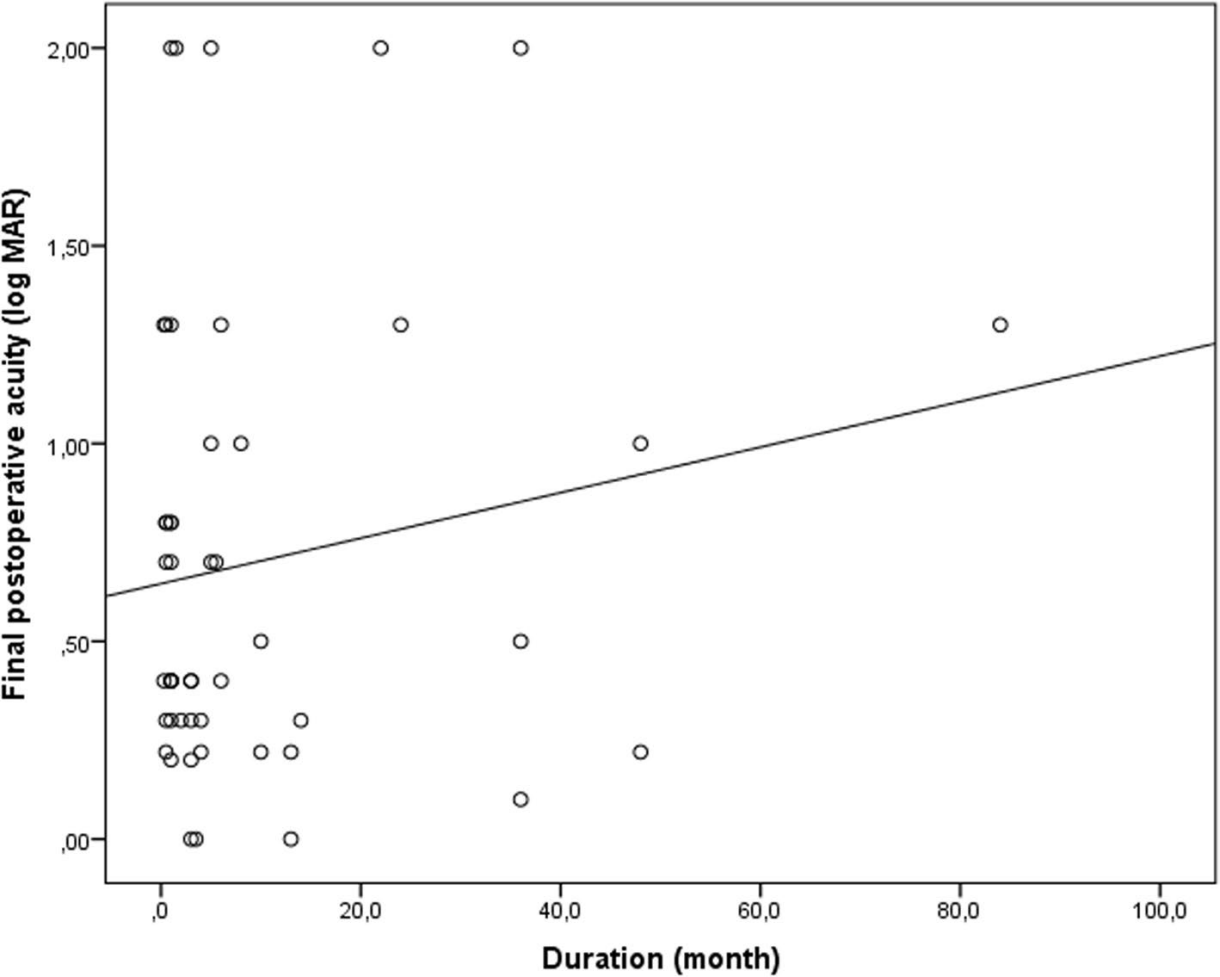
Pearson correlation analysis the duration between injury and cataract surgery (r = 0.163, *p* = 0.262)

In children undergoing cataract surgery, the mean duration between the time of primary injury and cataract surgery was 9.7 ± 15.9 months (n:49, range, 0.3–84 months; median, 3 months). We compared the final BCVA between eyes for which the time from the trauma injury and cataract surgery was less than 3 months and those operated on, more than 3 months after injury. The difference was not significant (*P* = 0.872) (Table 2). There was no correlation between this duration and the final BCVA (r = 0.163; *p* = 0.262) as shown in Fig. 2.

**Fig. 3:**
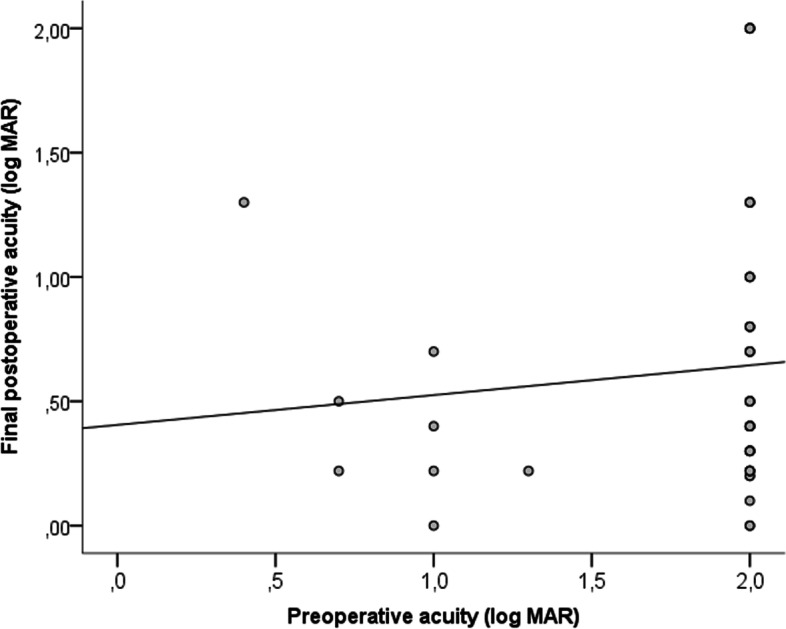
Spearman’s rank correlation analysis between the initial and final visual acuity (r = 0.132, *p* = 0.365)

The mean BCVA increased from 1.8 ± 0.42 logMAR before treatment to 0.61 ± 0.56 logMAR after treatment (Table 3). Spearman’s rank correlation analyses showed no correlation between the initial visual acuity and the final visual acuity (Fig. 3). There was no correlation between the initial and the final visual acuity (r = 0.132; *p* = 0.365) as shown in Fig. 3.

**Table 3:** Initial vs. final best corrected visual acuity (BCVA)

	Initial BCVA		Final BCVA	
Mean ± SD	1.8 ± 0.4		0.63 ± 0.56	
Range, min–max	0.4–2		0–2	
BCVA (logMAR)				
	n	%	n	%
> 0.3	–	–	20	36.4
0.7 to < 0.3	3	5.9	18	32.7
1.3 to < 0.7	5	10.2	15	27.3
≥ LP to 1.3	41	83.6	2	3.6
Total	49	100	55	100

Amblyopia was present in 34 (57.6%) patients, and strabismus was observed in 13 (22%) patients. The majority of children with amblyopia (30 [90%]) had penetrating trauma. A relationship was found between the age range at injury and the presence of amblyopia (*p* = 0.001), whereas no such relationship was evident with the presence of strabismus (*p* = 0.162) (Table 4). In age groups with amblyopia treatment, improvement in postoperative mean BCVA was better than initial vision (*p* < 0.001) (Table 5). Postoperative refractive rehabilitation was provided with glasses in 49 patients and contact lenses in 5 patients (1 for aphakia and 4 for refractive correction), whereas refractive rehabilitation was not needed in one patient.

**Table 4:** Amblyopia and strabismus presence rates according to age range at injury

Age range at injury (years)	Amblyopia presence		Strabismus presence	
	*n*	%	*n*	%
0–5	13	38.2	5	38.5
6–10	20	58.8	8	61.5
11–15	1	2.9	0	0

**Table 5:** Initial and final BCVA for amblyopia cases

Age range at injury (years)	Initial BCVA (logMAR)	Final BCVA (logMAR)	*p*
	Mean ± SD (min–max.)	Mean ± SD (n, min–max.)	
0–5	2 (*n* = 6, 2–2)	0.6 ± 0.27 (*n* = 13, 1–0.4)	< 0.001
6–10	1.93 ± 0.20 (*n* = 20, 2–1.3)	0.66 ± 0.52 (n = 20, 2–0.22)	< 0.001
11–15	2 (n = 1)	1.3 (n = 1)	–

Preoperative complication analyses revealed that the most common finding was pupil irregularity in 23 eyes (41.8%). Anterior and/or posterior synechiae were present in 12 eyes (21.8%), membrane formation in 11 eyes (20%), high intraocular pressure that could be controlled with medical treatment in 4 eyes (7.2%), and RD in 4 eyes.

(7.2%) (3 secondary to blunt trauma and 1 to penetrating trauma). All RD patients underwent vitreoretinal surgery. One patient with RD had severe learning difficulties and had suffered bilateral RD due to blunt trauma. This patient developed RD bilaterally again due to recurrent trauma after cataract surgery. Penetrating keratoplasty was performed in 3 eyes (5.45%) that had a large corneal scar developing secondary to penetrating trauma. There was also IOL decentralization in 2 eyes (3.63%), intravitreal hemorrhage in 1 eye (1.81%), and iris atrophy in 1 eye (1.81%) after cataract surgery.

## Discussion

Pediatric traumatic cataracts can result in amblyopia and strabismus by disturbing binocular vision, resulting in lifelong poor vision if not treated properly. Many studies have attempted to determine the factors that influence final vision; however, some uncertainties still exist [5, 6].

Analyses of the demographic features that influence traumatic cataract development and the results of treatment show that traumatic cataracts are more common in boys than in girls [6, 14–16]. Similarly, 71.2% of the patients in our study were males. This may be due to boys’ being more prone to experiencing trauma because of their activity level and tendencies toward outdoor play [15]. Injury was most common between the ages of 6 and 10 years, i.e., during the active play and school period, as reported by other studies [16, 17]. A relationship between the age range and final visual acuity was not found in this study, in line with previous reports [15, 18]. However, some studies have reported age and sex as significant variables with respect to the final visual acuity [15, 17].

As previously reported, penetrating trauma was more common than blunt trauma in our study [4, 17, 19, 20]. By contrast, Shah et al. and the other studies indicated that blunt trauma was more common than penetrating trauma [14, 21–23]. Some studies that have investigated the effects of trauma type on the final visual acuity have revealed that blunt traumas have a better prognosis than penetrating traumas [14, 18, 20–22, 24–26]. A relationship was not found between the type of trauma and the final visual acuity in the current study, similarly to that in two previous studies [4, 19]. With both trauma types, the presence of ocular pathologies besides cataracts, as well as other factors that could influence the final vision, may explain the different results reported in various studies.

Although the causes of ocular trauma vary globally because of different socioeconomic conditions and lifestyles, the most common cause of traumatic cataracts in our study was injury with a sharp object, a finding similar to that in previous works [27, 28]. We did not find an effect of the cause of trauma on the final BCVA, as in Shah et al. [17].

The most commonly involved area in our study was zone 1 (corneal) as in many pediatric trauma series [27–29]. Although zone 3 injuries may indicate a poor prognosis [29, 30], we did not find any effect of the injury site on final visual acuity. The reason for this could be the prompt primary repair of the injury to prevent complications and early interventions for any complications that might develop afterwards in our clinic. The low number of zone 2 and zone 3 injuries may also support our results.

The mean time between the primary injury and cataract surgery was 9.7 ± 15.9 months (median, 3 months), there was no correlation between the time from the trauma to cataract surgery and the final BCVA in accordance with previous studies [14, 17] .

PCO is more common in pediatric traumatic cataracts, which have more significant inflammation, than in non-traumatic cataracts [31, 32]. We observed PCO in all of the 13 patients older than 9 years who did not undergo PC + AV]. Significant evidence from this study and other studies suggests improved outcomes in patients who had undergone PC + AV, in terms of better final BCVA, compared with those who did not undergo this procedure [5, 19]. Therefore, the use of PC + AV for traumatic pediatric cataract surgery is likely to be helpful to avoid a high postoperative PCO rate (100% in our cases) and complications that could develop owing to YAG capsulotomy or surgical interventions to treat PCO in older children as well.

Another factor that could influence visual prognosis in pediatric traumatic cataracts is the time of IOL implantation. Many authors have reported the comparative advantages of primary or secondary IOL implantation [33, 34]. The IOL implantation was primary in 70.9% and secondary in 20%, whereas no IOL was used in the remaining 9.1% in this study. The time of IOL implantation had no effect on the final BCVA. Similarly, Rumelt et al. did not find a relationship between the time of implantation and a good BCVA [35]. The time of IOL implantation can be determined according to the child’s age and the degree of trauma. Good visual acuity may be obtained with proper indication, regular follow-up, and appropriate optic rehabilitation. The time of IOL implantation by itself may not be a variable for final vision in this aspect. The IOL type and IOL insertion location during surgery differed according to the location and type of trauma as well as the capsular and zonular integrity of the lens. A effect of whether the IOL was placed into the sulcus or capsular bag on the final BCVA was not found in this study, as in a previous series [14, 36].

The ratio of children with a visual acuity of 0.3 logMAR or better following traumatic cataract was 36.4% in our study. In an another study the ratio of those with a final visual acuity of 0.3 logMAR or better (Snellen 6/12) has been reported to be 34.4% in their long-term follow-up [37]. Also in our study the correlation was not observed between the initial visual acuity and the final visual acuity. However many studies that consider the initial visual acuity in traumatic pediatric cataracts as a prognostic factor for final visual acuity [3, 14, 29]. We believe that this result was influenced by our study being based on data obtained over a 10-year period, the complications seen during follow-up being managed promptly and correctly, amblyopia being prevented as much as possible, and the follow-ups being performed by the same ophthalmologist over many years. Gogate et al. [15] and Jinagal et al. [19] also did not find a significant correlation between preoperative vision and final vision in their studies covering a long time period.

Optic rehabilitation and amblyopia treatment must be started as soon as possible with regular follow-up and close cooperation of the family in the pediatric age group with a traumatic cataract, where an amblyopia risk exists [38]. The presence of amblyopia and success of treatment are important factors regarding the visual prognosis in these children [1, 3, 19, 35]. We believe that the results were better with appropriate optic rehabilitation, effective patching, and regular follow-up for amblyopic eyes observed in this study. The lack of a relationship between the presence of strabismus and both the age group and final vision could be related to the development of binocular vision before the trauma in most patients [39].

The limitations of this study were its retrospective nature and the relatively high rate of complicated cases in the series, as it was conducted at a tertiary referral center. However, well documented and standardized structure of the data from a single center with a remarkable number of pediatric patients who presented with ocular trauma is one of the strongest aspects of our manuscript along with the considerably long term patient follow-up periods. A few reports in similar patient populations exist in the literature. In one of these studies, Hilely et al. [40] reported no relation between final visual acuity and time interval between injury and IOL implantation. However, this study did not report any effects of posterior capsulotomy (PC) and anterior vitrectomy (AV) during cataract surgery. In another study [19] with a similar long-term follow-up period has addressed the problem of traumatic pediatric cataracts with specific reference to the approaches for improvements in the final best corrected visual acuity (BCVA) and the importance of combining PC + AV with lens aspiration with or without IOL implantation during cataract surgery.

Similar to these reports, our analyzes revealed that good final visual acuity (better than 0.5 logMAR, in accordance with the WHO guidelines) could be achieved, regardless of the child’s age, sex, initial visual acuity, type, cause and zone of injury, and time of IOL implantation. The only factors affecting visual acuity in the long term period were found to be PC + AV in pediatric traumatic cataracts.

## Conclusions

Results from the current retrospective study suggest that good visual results, as defined by WHO criteria, can be achieved by using PC and AV along with lens aspiration and IOL implantation in children with traumatic cataracts, followed by effective amblyopia treatment and regular follow-up and monitoring. However, more comprehensive and prospective studies are needed to confirm this approach of amblyopia treatment.

## Supplementary Information

**Additional file 1.** Data Sheet 1: Raw Data of the Manuscript.

## Data Availability

All data generated or analyzed in this study are included in this published article and supplementary information files.

## Abbreviations

AV: Anterior vitrectomy
BCVA: Best corrected visual acuity
PC: Posterior capsulotomy
PCO: Posterior capsule opacification
RD: Retinal detachment
TSF: Trans-scleral fixation
PPC: Primary posterior vitrectomy,
PCIOL: Posterior chamber intraocular lens

## References

[1] Shah M, Shah S, Upadhyay P, Agrawal R (2013). Controversies in traumatic cataract classification and management: a review. Can J Ophthalmol.

[2] Khokhar S, Agarwal T, Kumar G, Kushmesh R, Tejwani LK (2012). Lenticular abnormalities in children. J Pediatr Ophthalmol Strabismus.

[3] Reddy AK, Ray R, Yen KG (2009). Surgical intervention for traumatic cataracts in children: epidemiology, complications, and outcomes. J AAPOS.

[4] Shah MA, Shah SM, Appleware AH, Patel KD, Rehman RM, Shikhange KA (2012). Visual outcome of traumatic cataract in pediatric age group. Eur J Ophthalmol.

[5] Verma N, Ram J, Sukhija J, Pandav SS, Gupta A (2011). Outcome of in-the-bag implanted square-edge polymethyl methacrylate intraocular lenses with and without primary posterior capsulotomy in pediatric traumatic cataract. Indian J Ophthalmol.

[6] Krishnamachary M, Rathi V, Gupta S (1997). Management of traumatic cataract in children. J Cataract Refract Surg.

[7] Buckley EG, Klombers LA, Seaber JH, Scalise-Gordy A, Minzter R (1993). Management of the posterior capsule during pediatric intraocular lens implantation. Am J Ophthalmol.

[8] Pieramici DJ, Sternberg P, Aaberg TM, Bridges WZ, Capone A, Cardillo JA (1997). A system for classifying mechanical injuries of the eye (globe). Am J Ophthalmol.

[9] Pararajasegaram R (2002). Importance of monitoring cataract surgical outcomes. Community Eye Health J.

[10] Drews-Botsch CD, Hartmann EE, Celano M, Group IATS (2012). Predictors of adherence to occlusion therapy 3 months after cataract extraction in the Infant Aphakia Treatment Study. J AAPOS.

[11] The Pediatric Eye Disease Investigator Group (2003). A randomized trial of prescribed patching regimens for treatment of severe amblyopia in children. Ophthalmology..

[12] Repka MX, Beck RW, Holmes JM, Birch EE, Chandler DL, Cotter SA (2003). A randomized trial of patching regimens for treatment of moderate amblyopia in children. Arch Ophthalmol (Chicago, Ill: 1960).

[13] Scheiman MM, Hertle RW, Beck RW, Edwards AR, Birch E, Cotter SA (1960). Randomized trial of treatment of amblyopia in children aged 7 to 17 years. Arch Ophthalmol (Chicago, Ill).

[14] Gurung G, Bajracharya K (2020). Visual outcome of pediatric traumatic cataract in Lumbini Eye Institute, Bhairahawa, Nepal. Nepal J Ophthalmol.

[15] Gogate P, Sahasrabudhe M, Shah M, Patil S, Kulkarni A (2012). Causes, epidemiology, and long-term outcome of traumatic cataracts in children in rural India. Indian J Ophthalmol.

[16] Burgos-Elías VY, Marroquín-Sarti MJ, Zimmermann-Paiz MA, Rivas AMO, Quezada-del Cid NC (2018). Traumatic cataract surgery in pediatric patients. Experience in a site. Arch Argent Pediatr.

[17] Shah M, Shah S, Shah S, Prasad V, Parikh A (2011). Visual recovery and predictors of visual prognosis after managing traumatic cataracts in 555 patients. Indian J Ophthalmol.

[18] Khokhar S, Gupta S, Yogi R, Gogia V, Agarwal T (2014). Epidemiology and intermediate-term outcomes of open-and closed-globe injuries in traumatic childhood cataract. Eur J Ophthalmol.

[19] Jinagal J, Gupta G, Gupta PC, Yangzes S, Singh R, Gupta R (2019). Visual outcomes of pediatric traumatic cataracts. Eur J Ophthalmol.

[20] Xu YN, Huang YS, Xıe LX (2013). Pediatric traumatic cataract and surgery outcomes in eastern China: a hospital-based study. Int J Ophthalmol.

[21] Shah MA, Shah SM, Gosai SR, Gupta SS, Khanna RR, Patel KB (2018). Comparative study of visual outcome between open-and closed-globe injuries following surgical treatment of traumatic cataract in children. Eur J Ophthalmol.

[22] Puodžıuvıenė EE, Jokūbauskienė G, Vieversytė M, Asselineau K (2018). A five-year retrospective study of the epidemiological characteristics and visual outcomes of pediatric ocular trauma. BMC Ophthalmol.

[23] Kınorı M, Tomkins-Netzer O, Wygnanski-Jaffe T, Ben-Zion I (2013). Traumatic pediatric cataract in southern Ethiopia—results of 49 cases. J AAPOS.

[24] Du Y, He W, Sun X, Lu Y, Zhu X (2018). Traumatic cataract in children in eastern China: Shanghai pediatric cataract study. Sci Rep.

[25] Zhu L, Wu Z, Dong F, Feng J, Lou D, Du C (2015). Two kinds of ocular trauma score for paediatric traumatic cataract in penetrating eye injuries. Injury..

[26] Shah MA, Shah SM, Patel KD, Shah AH, Pandya JS (2014). Maximizing the visual outcome in traumatic cataract cases: the value of a primary posterior capsulotomy and anterior vitrectomy. Indian J Ophthalmol.

[27] Ilhan HD, Bilgin AB, Cetinkaya A, Unal M, Yucel I (2013). Epidemiological and clinical features of paediatric open globe injuries in southwestern Turkey. Int J Ophthalmol.

[28] Liu X, Liu Z, Liu Y, Zhao L, Xu S, Su G (2014). Determination of visual prognosis in children with open globe injuries. Eye..

[29] Acar U, Tok OY, Acar DE, Burcu A, Ornek F (2011). A new ocular trauma score in pediatric penetrating eye injuries. Eye..

[30] Knyazer B, Levy J, Rosen S, Belfair N, Klemperer I, Lifshitz T (2008). Prognostic factors in posterior open globe injuries (zone-III injuries). Clin Exp Ophthalmol.

[31] Trivedi RH, Wilson ME (2015). Posterior capsule opacification in pediatric eyes with and without traumatic cataract. J Cataract Refract Surg.

[32] Sun N, Trivedi RH, Wilson ME (2012). Posterior capsule opacification (PCO) in pediatric eyes with and without traumatic cataract surgery with in-the-bag single-piece hydrophobic acrylic IOL implantation. J AAPOS.

[33] Yardley AM, Ali A, Najm-Tehrani N, Mireskandari K (2018). Refractive and visual outcomes after surgery for pediatric traumatic cataract. J Cataract Refract Surg.

[34] Sen P, Shah C, Sen A, Jain E, Mohan A (2018). Primary versus secondary intraocular lens implantation in traumatic cataract after open-globe injury in pediatric patients. J Cataract Refract Surg.

[35] Rumelt S, Rehany U (2010). The influence of surgery and intraocular lens implantation timing on visual outcome in traumatic cataract. Graefes Arch Clin Exp Ophthalmol.

[36] Zhao Y, Gong X, Zhu X, Li H, Tu M, Coursey TG, et al. Long-term outcomes of ciliary sulcus versus capsular bag fixation of intraocular lenses in children: an ultrasound biomicroscopy study. PLoS One. 2017;12. 10.1371/journal.pone.0172979. 10.1371/journal.pone.0172979 PMC5354427 28301497

[37] Adlina AR, Chong YJ, Shatriah I (2014). Clinical profile and visual outcome of traumatic paediatric cataract in suburban Malaysia: a ten-year experience. Singap Med J.

[38] Toro MD, Bremond-Gignac D, Brézin AP, Cummings AB, Kemer OE, Kermani O (2022). COVID-19 outbreak and increased risk of amblyopia and epidemic myopia: ınsights from EUROCOVCAT group. Eur J Ophthalmol.

[39] Magli A, Forte R, Carelli R, Magli G, Esposito F, Torre A (2016). Long-term follow-up after surgery for congenital and developmental cataracts. Semin Ophthalmol.

[40] Hilely A, Leiba H, Achiron A, Hecht I, Parness-Yossifon R (2019). Traumatic cataracts in children, long-term follow-up in an Israeli population: a retrospective study. Isr Med Assoc J.

